# How qualitative studies can strengthen occupational health research

**DOI:** 10.5271/sjweh.3943

**Published:** 2021-03-01

**Authors:** Cécile RL Boot, Astrid R Bosma

**Affiliations:** Amsterdam UMC, VU University, Amsterdam Public Health Research Institute, Department of Public and Occupational Health, Amsterdam, The Netherlands; Radboud University, Behavioural Science Institute, Nijmegen, The Netherlands

The *Scandinavian Journal of Work, Environment and Health* (SJWEH) has a strong reputation in publishing high quality studies using quantitative research methods in the field of occupational health. Studies using qualitative research methods, however, are rarely published in the Journal. In this editorial, we explain how qualitative studies can contribute to the field of occupational health research *and* to SJWEH’s aim to promote high quality and impactful research in the field of occupational and environmental health and safety and increase knowledge through scientific publications.

SJWEH has its origin in occupational medicine, toxicology and epidemiology, providing answers to questions about causal mechanisms between workplace exposures and health (1). The impact of these causes in the environment on health can be quantified in occupational epidemiological studies. Addressing environmental causes to improve health requires collective action of the (work) environment. Over the years, the field of occupational health – as well as the Journal – has broadened its focus to capture the complex relationship between the larger work environment and health in general (2). The multitude of etiologies of current occupational and environmental health problems requires more complex interventions (3). Studying the impact of human behavior and its interaction with the environment became more important (4). Insights from social sciences are indispensable to understanding human behavior, for example, studying the eating habits of shift workers (5). With this broader focus, research designs other than quantitative ones have emerged, such as qualitative research designs. With its interpretative and inductive approach, qualitative research is aimed at understanding people’s experiences, behaviors and interactions by interpreting a subjective and constructed reality, while quantitative research is aimed at investigating and explaining phenomena, assuming an objective reality (6–10). Qualitative research can provide insights about new phenomena, experiences or processes. Furthermore, qualitative research is valuable when the aim is to describe the larger social context in relation to the topic of interest (6). For example, qualitative research could aid in better understanding how workers with, eg, cancer or chronic conditions are successful in staying at or returning to work (11–15). Experience and interpretation of symptoms and limitations can provide information on a wide range of barriers and facilitators of working with chronic conditions that are difficult to capture in quantitative data. Such data can only be retrieved from an in-depth analysis of processes and context.

To better understand the potential of qualitative research, it is helpful to reflect on its historical development, which Pertti Alasuutari comprehensively described (16). Qualitative research developed from an interpretative approach that has its roots in philosophy, anthropology and history and goes back to the nineteenth century (6). Sociologists were the first to systematically apply an interpretative approach for the purpose of scientific research (16). Until then, positivism was the dominant paradigm in science, including in sociology (16, 17). Positivism is a philosophical theory positing that knowledge is derived from empiricism by taking a reductionist approach (17). In a reductionist approach, (social) reality is reduced to causal chains of predefined variables that are fit into models.

Max Weber (1864–1920) was one of the key pioneers of sociology who criticized positivism in sociology (16). His research focused on norms and values. He proposed an interpretative understanding of actions rather than taking a reductionist or deterministic approach. This is what is now referred to as qualitative research. An interpretative approach is useful to generate new theory.

From the above, it may seem as if the positivism paradigm is used in quantitative research only, but that is not true. Although the inductive approach is most often used in qualitative research, qualitative designs can also be applied to test existing theory by applying a more deductive than inductive approach to verify or falsify theory.

Different study designs can provide complimentary perspectives. Qualitative research is the best choice when the context and its complex interactions with human behavior play an essential role in the studied phenomenon, for example, to understand the nature of relationships between events in their social or economic context or to explore different experiences (eg, 10–13). Qualitative research designs are needed to capture elements that cannot (yet) be captured in numbers but are essential to a better understanding. A mixed-methods design, in which quantitative and qualitative research methods inform and complement each other, has the potential to combine the best of both worlds (eg, 14,18).

For SJWEH, high external validity – generalizability to other contexts – is an important indicator of quality and a strong determinant of acceptance for publication. As qualitative studies aim for a rich description of a specific context, generalization is not an aim of high quality qualitative research. For qualitative studies, external validity depends on transferability to another context. Elements of transferability are the description of the context for the purpose of transferability, a maximum variation in the sample, and comparison with literature (19). In essence, transferability relates to how the knowledge generated in qualitative studies contributes to a better understanding of how the (work) environment affects health. This may comprise new insights into needs or experiences of workers who are facing new problems (eg, 11), perceived barriers related to a complex process (eg, 12), or a better understanding of why a workplace intervention was not as successful as expected (eg, 15).

So how to compose a high quality manuscript of a qualitative study? Not surprisingly, many criteria for high quality studies are similar for both types of research designs, although the operationalization differs. The consolidated criteria for reporting qualitative research (COREQ) 32-item checklist was developed as a qualitative counterpart of the CONSORT statement for quantitative studies (20). The COREQ32 checklist aims to improve the quality of reporting of qualitative studies, and strives for greater recognition of qualitative research to be considered as equal to quantitative research (20). The checklist consists of 32 criteria relating to (i) research team and reflexivity; (ii) study design; and (iii) analysis and findings. Adhering to this guideline is strongly recommended as it will likely increase the methodological quality of a manuscript.

Following are five recommendations for a qualitative study to be published in the Journal.

1. In qualitative designs, researchers are much more involved with the participants. A reflection on how the involvement of the researchers, based on their personal characteristics might have influenced the results should be included in the manuscript.
2. In the introduction, a case should be made for why qualitative research was the most appropriate route to answer the research question. For example, explain why it is important that we know experiences of professionals about return to work of cancer patients (11). Describe how this will contribute to what is already known.
3. In the methods section, the theoretical framework, setting and recruitment of participants and analyses should be carefully described. It is important to keep in mind that most readers of SJWEH will likely have more experience with quantitative than qualitative research designs. Checks and balance ensuring a high quality procedure should be included, for example: a dual coding procedure in which two researchers have coded transcripts independently to prevent bias; a clear description of how the codes were categorized into themes, or a member check to ensure the participants supported the summary of the interviews in which they took part.
4. In the discussion section, a case should be made for how the findings of the study can be transferred to other contexts and how they generally improve the understanding of phenomena in the field of occupational health research.
5. Apply the COREQ32 checklist to your qualitative study (20).

We warmly invite you to submit your high quality manuscripts with a qualitative research design to SJWEH!
